# A Water Soluble 2-Phenyl-5-(pyridin-3-yl)-1,3,4-oxadiazole Based Probe: Antimicrobial Activity and Colorimetric/Fluorescence pH Response

**DOI:** 10.3390/molecules27061824

**Published:** 2022-03-11

**Authors:** Rosita Diana, Ugo Caruso, Luigi Di Costanzo, Simona Concilio, Stefano Piotto, Lucia Sessa, Barbara Panunzi

**Affiliations:** 1 Department of Agriculture, University of Naples Federico II, Via Università, 100, 80055 Portici, Italy; rosita.diana@unina.it (R.D.); luigi.dicostanzo4@unina.it (L.D.C.); barbara.panunzi@unina.it (B.P.); 2 Department of Chemical Sciences, University of Naples Federico II, Strada Comunale Cinthia, 26, 80126 Napoli, Italy; 3 Department of Pharmacy, University of Salerno, Via Giovanni Paolo II, 132, 84084 Fisciano, Italy; sconcilio@unisa.it (S.C.); piotto@unisa.it (S.P.); lucsessa@unisa.it (L.S.)

**Keywords:** pH-responsive probe, naked-eye colorimetric response, naked-eye fluorescence response, antibacterial activity, molecular dynamics studies on probe–cell membrane interactions

## Abstract

The growing demand of responsive tools for biological and biomedical applications pushes towards new low-cost probes easy to synthesize and versatile. Current optical probes are theranostic tools simultaneously responsive to biological parameters/analyte and therapeutically operating. Among the optical methods for pH monitoring, simple small organic molecules including multifunctional probes for simultaneous biological activity being highly desired by scientists and technicians. Here, we present a novel pH-responsive probe with a three-ring heteroaromatic pattern and a flexible cationic chain. The novel molecule shows real-time naked-eye colorimetric and fluorescence response in the slightly acidic pH range besides its excellent solubility both in the organic phase and in water. In addition, the small probe shows significant antibacterial activity, particularly against *Escherichia coli*. Single-crystal X-ray study and density functional theory (DFT) calculations rationalize the molecule spectroscopic response. Finally, molecular dynamics (MD) elucidate the interactions between the probe and a model cell membrane.

## 1. Introduction

Optical probes offer in situ observation, real-time and fast-response monitoring, sensitivity, selectivity [1,2,3], and even by the non-invasive approach required for living tissues [4,5,6]. Colorimetric/fluorescent tools revolutionized the ability to probe biological dynamics at the cellular level on several platforms: small-molecule dyes, metal complexes, and fluorescent proteins [7,8,9,10,11,12,13,14,15,16,17,18,19,20]. The oldest family of photoluminescence (PL) sensors encompass small organic molecules [14,19,21,22,23,24,25,26,27,28,29], still sought-after but in a different perspective. Today’s demand for responsive multifunctional tools pushes to collaborative work between organic synthesis and biological and biomedical requirements. Current optical probes are theranostic tools simultaneously responsive to biological parameters (pH, for instance) and therapeutic featuring [30,31,32,33,34]. 

As for the pH parameter, simple small organic molecules still focus the attention of scientists and technicians [35]. Specifically, from the key role of lysosomes in cellular necrosis [36] comes the need for acidic pH-activatable probes monitoring acidic pH (5.0–6.0) as opposed to more basic cytoplasm’s pH (≈7.4) [4,37,38,39]. Desirable requirements for pH monitoring in living tissue are good water solubility and noticeable PL signal in an aqueous environment. Furthermore, it should be considered that many PL tools suffer from the quenching effect in water and in a diluted solution known as ACQ (aggregation caused quenching effect) [40,41,42,43,44,45]. In addition, probes for biomedical and biological purposes should have good cell membrane permeability to cross plasma with a minimal perturbation [46,47], due to having hydrophilic and a hydrophobic character [46,48]. Fluorescent probes able to monitor pH by turn-on or turn-off fluorescence/colorimetric signal (a spectral shift and/or a change in signal intensity) can be useful tools in monitoring abnormal cells or different intracellular regions, as confirmed by recent articles [49,50].

Nitrogen and oxygen-based aromatic rings can play the role of donors, acceptors and even bridges for the electronic density. In addition, many heterocycles behave as potential pH-sensitive fragments. Specifically, the presence of oxadiazoles and pyridinyl rings can provide extremely responsive compounds: many 1,3,4-oxadiazole derivatives showed their potential for an array of bioactivities [51] and therapeutical application [52,53,54]. Due to their ability to undergo various chemical functionalization [55], 1,3,4-oxadiazole can bind targeted metabolic analytes [51] and are employed in synthesizing pharmaceutical drugs, anticancer agents [56,57], and pH probes for living cells [58].

Used as a bridge between the pyridinyl ring and a substituted phenyl ring, 1,3,4-oxadiazole can result in biologically active molecules. There are some examples in the literature where probes with both characteristics of antimicrobial activity and pH responsivity are used [59,60,61]. Specifically, different substituted phenyl-5-(pyridin-3-yl)-1,3,4-oxadiazole were studied for their activity against common bacteria and fungi [62,63,64,65] as *Bacillus subtilis*, *Staphylococcus aureus*, *Pseudomonas aeruginosa*, *Proteus vulgaris*, *Klebsiella pneumoniae*, *Escherichia coli*, and *Candida albicans*. Interestingly, the presence of nitro and/or hydroxy substituents on the phenyl ring directs and, in some cases, increases the molecule’s activity. On the other hand, the three-ring scaffold is scarcely soluble in an aqueous environment and rarely explored from the spectroscopic point of view [66,67].

**Scheme 1:**
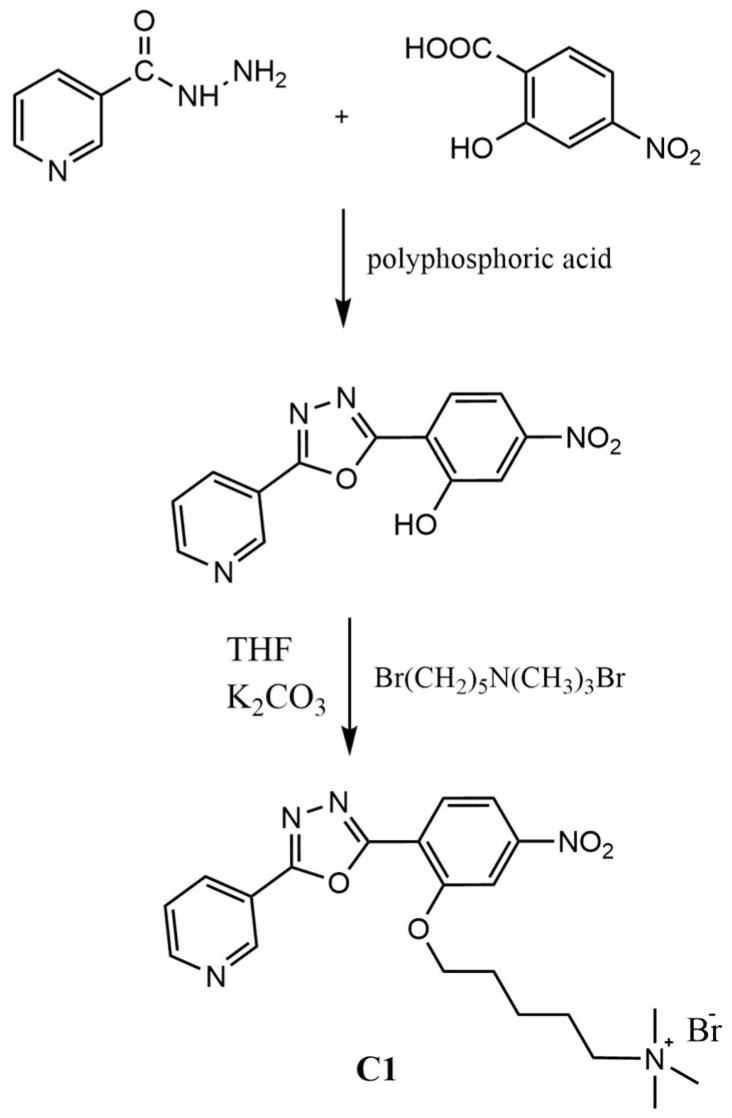
Schematic representation of the two-step synthetic route to the target compound C1.

Here, we design a targeted modification of a substituted phenyl-5-(pyridin-3-yl)-1,3,4-oxadiazole, which proved [60] an interesting ability against *Escherichia coli.* The introduction of an amphiphilic chain on the aromatic scaffold demonstrated a good protocol to increase water solubility, preserving the antimicrobial activity. Despite the nitro group typically depressing fluorescence, the easy-to-make small probe C1 (in Scheme 1) is emissive in pure water at slightly acidic pH. The pyridinyl ring being the primary pH-responsive site contributes to the probe color switch (from colorless to yellow) and fluorescence quenching, observed by turning the pH from the value of 5.5 to 6.5. Single-crystal X-ray analysis gave information about the structure of C1 and was employed as a basis for density functional theory (DFT) calculations, achieving a rationalization [68,69] of the spectroscopic response.

## 2. Results and Discussion

### 2.1. Design and Spectroscopic Behavior of C1

The molecule C1 was obtained by a two-steps route (cyclization and functionalization of the hydroxyl group). The main pH-sensitive site was expected to be the nitrogen atom group on the pyridinyl ring (pK_a_ ≈ 5.5). On the other hand, the 1,3,4-oxadiazole ring can add a potential contribution in the acidic range (pK_a_ ≈ 3.8). The jointed bent shaped architecture of the aromatic part and the charged side chain promote solubility both in water and non-aqueous environment, as desirable for the efficient delivery into living cells intracellular compartments [22]. The probe is stable in distilled water and soluble up to 10 mM. C1 is also soluble in common polar organic solvents such as chloroform, dioxane, acetone, ethanol, *N*,*N*-dimethylformamide, and DMSO, solubility increasing with the polarity of the solvent. 

Compared to its precursor 5-nitro-2-(5-(pyridin-3-yl)-1,3,4-oxadiazol-2-yl)phenol, C1 is soluble and emissive in water. As expected [70], the chain increases the affinity for water. It makes the molecule fluorescent due to the implemented emission related to the electrostatic repulsions between the cationic chains [71,72,73]. Therefore, the C1 molecule is a light-yellow non-emissive crystalline powder in the solid-state. Dissolved in distilled water (pH = 6.25) C1 is quite colorless, and its light blue emission can be ascribed to ACQ (aggregation caused quenching) effect [40,41,43].

**Figure 1:**
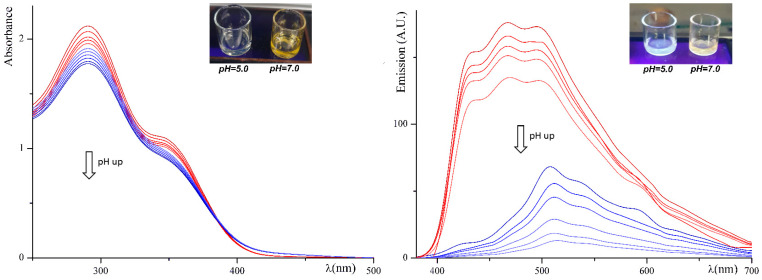
UV–visible spectral titration of C1 1.00 mM in water using Britton–Robinson buffers from pH = 4.0 to pH = 9.0 by gradients of 0.5 pH units (on the left). Fluorescence spectral titration of C1 1.00 mM in water using Britton–Robinson buffers from pH = 4.0 to pH = 9.0 by gradients of 0.5 pH units (on the right), excitation wavelength of 280 nm. In the insets: the same water solutions used to register the absorbance and emission spectra in natural light (on the left) and under a standard UV-lamp at 254 nm (on the right) at two different pH values.

C1 underwent a spectroscopic analysis by absorption and emission spectroscopy. The same buffered water solutions were used for titration experiments (Figure 1) and the naked-eye analysis in natural light and under a UV lamp operating at 254 nm. In the insets in Figure 1, it can be appreciated the real-time naked-eye response as a colorimetric “turn-on” and a fluorescence “turn-off” effect over a slightly acidic pH value (above 6). Specifically, the solutions appear colorless up to pH = 5.5, turning to light-yellow starting from pH = 6.5. A fluorescence response is observable as well. The colorless solutions photographed under visible light are whitish emissive and more intense with respect to the yellow-colored solutions. We experimented different solvents, enclosed mixture water/DMSO, recording similar trends in the emission/absorbance maxima. In organic solvents such as ethanol, acetone the emission is more intense, and the yellow color of the solution over pH = 6.5 is less appreciable. In DMSO, the emission is improved due to the viscosity-activated fluorescence booster effect [74,75,76]. In water, the emission is less intense but perceivable. As expected, PLQYs in water/DMSO mixtures (1:1 and 2:1) are intermediate respect to pure water and pure DMSO.

As a comparison, the absorption and emission maxima and PLQYs for both water and DMSO buffered solutions recorded in the slightly acidic and neutral environment are reported in Table 1. The two buffered DMSO solutions can be considered as “protonated C1 dissolved in DMSO” (pH = 5.0 buffer) and “unprotonated C1 dissolved in DMSO” (pH = 7.0 buffer). In the pH range of greatest interest (from pH =5.0 to pH = 7.0) PLQY measured in water decreases to almost one eighth and in DMSO decreases to nearly a quarter (see Table 1). Although elaborate systems show higher quantitative responses [77], recent literature provides small organic pH probes able to distinguish between normal and tumors cells with comparable on-off fluorescence response [26].

The absorption and emission titration experiments performed in 1.00-mM buffered water solutions show two different patterns switching between pH = 5.5 and pH = 6.5, (Figure 1) in a general on-off behavior. In the absorption titration, most of the broad humped absorption band is located before the visible region (before 390 nm), and the pattern is preserved up to pH = 6.0. Starting from the spectrum recorded at pH = 6.5, the part of the band located over 390 nm (responsible for the yellow color of the solutions) increases. The absorbance curves reported in Figure 1 (left side) were recorded as the pH increases from 4.0 to 9.0 by gradients of 0.5 pH units. A close up of the Figure 1 is reported in Supplementary Materials, Figure S2 (see Supplementary Materials). It is very evident that the behavior of C1 changes in the range of pH 6.0–6.5. In fact, there is an inversion of the absorption curves between these two values (at about 390 nm). The response of the acidic block (above) is quite distinct from the basic block (below), and the turn-off is between 6.0 and 6.5. 

Analogous behavior is detected in the fluorescence titrations (Figure 1, right side) recorded on aqueous buffered solutions by increasing pH from 4.0 to 9.0 by gradients of 0.5 pH units. The general emission pattern in the acidic environment presents a three-peaked band centered at about 467 nm. A gradual decrease in intensity is recorded as pH increases from 4.0 to 9.0 by gradients of 0.5 pH units. Starting from the solution buffered at pH = 6.5, the band decreases rapidly and undergoes a red-shift to 508 nm. The colorimetric and fluorescence behavior are reversible within the examined pH range. The water solutions are stable, and the spectra are replicable after two weeks at room temperature. Contrarily, the replicability is scarce at extremely acidic or basic pH values, and the solutions appear slightly turbid after some days at room temperature. 

### 2.2. Antibacterial Activity 

As C1 is a highly water-soluble molecule, we tested the antimicrobial activity against the most common polluting bacteria of aqueous media. The Coliform bacteria, whose presence is a key indicator of water quality, were evaluated. The molecule C1 shows significant antibacterial activity, especially against *Escherichia coli*, and similar to its precursor, 5-nitro-2-(5-(pyridin-3-yl)-1,3,4-oxadiazol-2-yl) phenol [65]. Specifically, following the standard UNI EN ISO 8199:2018 and the standard UNI EN ISO 9308-1:2017, the colony-forming units (CFU) were evaluated at different concentrations of C1 stock solutions. The sample obtained by adding 2.50 mL of the stock solution of C1 (10 mM) to 50 mL of specific agar medium showed 10 CFU/100 mL respect to the standard reference (20 CFU/100 mL), i.e., about 50% inhibition of CFU. The 90% CFU abatement was found by treating 50 mL of the specific agar medium with a stock solution containing an amount of 0.05 mmol of C1.

### 2.3. X-ray Structural Characterization of C1

**Figure 2:**
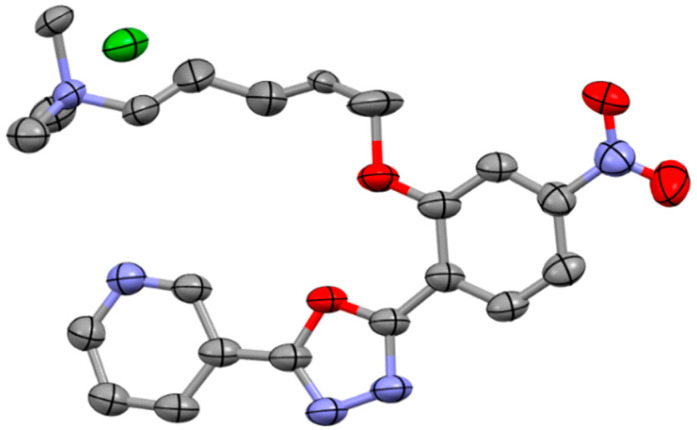
Molecular structure of C1•Br. Thermal ellipsoids are drawn at 50% probability level. Atoms are color-coded as follows: carbon (gray), oxygen (red), nitrogen (blue), bromine (green).

**Figure 3:**
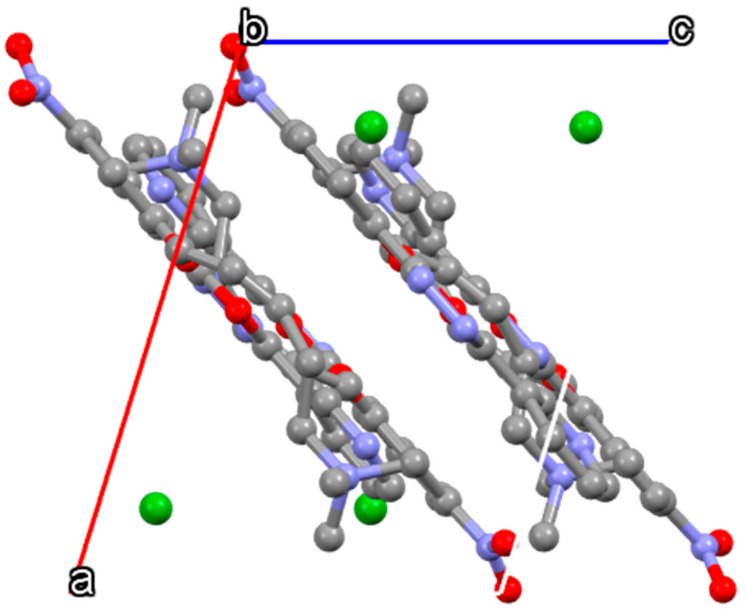
Crystal packing of C1•Br along b axis direction.

Crystals of C1 were obtained by slow evaporation at room temperature and are characterized by a bright yellow color (see Section 2.2). The asymmetric unit of C1 contained one bromide ion counterbalancing the positive charge of the C1 molecule and ensuring an overall electroneutrality. The U-shaped C1 molecule, shown in Figure 2, was characterized by the essentially coplanar three cycles of the aromatic moiety with a slight rotation of its terminal pyridinium group. The oxygen atom group of the 1,3,4-oxadiazole cycle and the oxygen atom group of the butyl ester were in the same plane. The nitro group was also slightly rotated out of the plane of the aromatic group. The aliphatic methyl groups of the butyl ester and its associated trimethyl ammonium group lay alongside the -Po segment in a linear fashion (Figure 3), making C1 molecule an overall nearly flat molecule. The molecules of C1 were packed with a head-to-tail antiparallel arrangement and self-assembled into π-stacked columns along the **b** axis, as shown in Figure 3. The packing was characterized by several short (~3.3 Å) π–π interactions involving the main plane of the aromatic moiety. This orientation was stabilized by several intermolecular Van-der Waals interactions involving the aliphatic groups of the n-butyl chains. 

### 2.4. Molecular Dynamics Simulations

**Figure 4:**
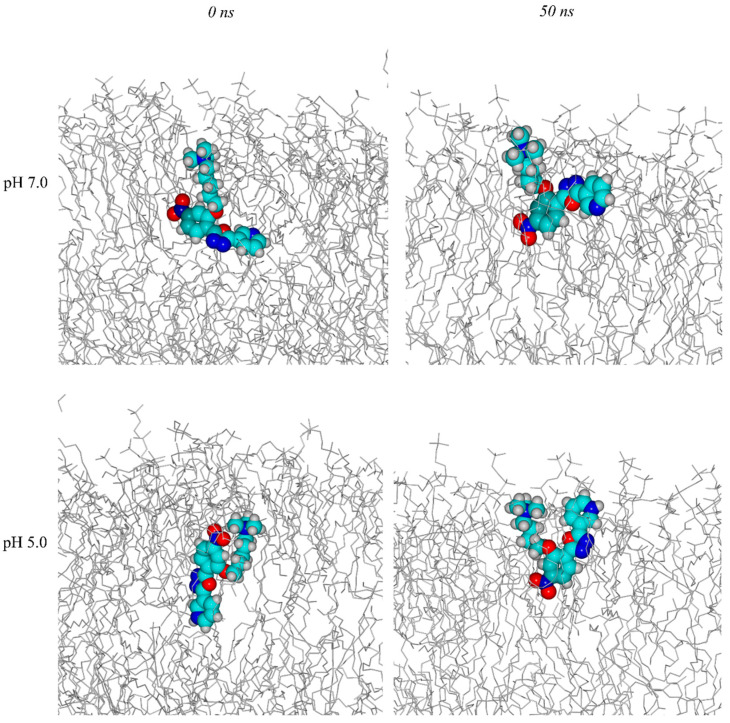
C1 in POPC membrane at 0 ns, at pH = 7.0 and pH = 5.0; at 50 ns, at pH = 7.0 and pH = 5.0.

We determined the ability of C1 binding and crossing of the lysosomal membrane and the degree of bilayer perturbation, as consequence of lipidic interaction, by molecular dynamics simulations. We used as a model a POPC membrane. The initial state and the state at the end of 50 ns of simulation for the protonated and deprotonated form (supposed the main form at pH = 5.0 and the main form at pH = 7.0) are shown in Figure 4.

**Figure 5:**
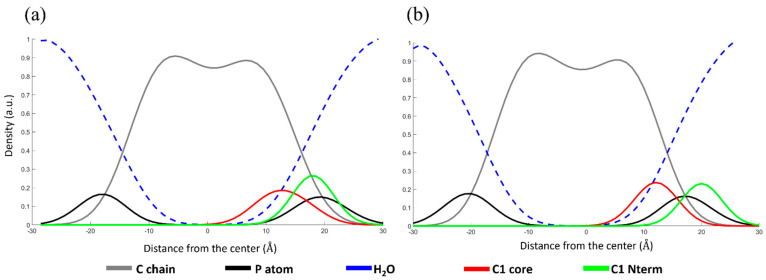
Density profile (**a**) at pH = 5.0, (**b**) at pH = 7.0. The chains and the heads of POPC are shown in gray and black lines, respectively. Dashed blue line represent water molecules. C1 aromatic core and the C1 N terminal are shown in red and green, respectively.

For a small molecule like C1, an all-atoms dynamic of 50 ns is sufficient to determine the membrane/water partition coefficient. The density profile indicates the distribution of atoms along the bilayer of the membrane. The density profile (see Figure 5) shows a weak orientation of C1, both protonated and deprotonated, with the charged head at the membrane/water interface and the aromatic core inside. Although pH cannot be defined in an anhydrous medium as a lipid bilayer, simulations of the different forms indicate similar behavior. The graphs in Figure 5a,b show the distribution of the different membrane components along with the normal phospholipid layer. The dashed blue line represents water; the solid gray line represents the carbon atoms of the phospholipid chain. The black line represents the P atoms of the phospholipid heads. The red and green lines represent the core and the tail of probe C1. 

The probe is located with the tricyclic core below the phospholipid heads and the tail at the water-membrane interface. Furthermore, we can infer from the amplitude of the red curve that, at pH = 7.0, the probe folded parallel to the membrane surface, whereas, at pH = 5.0, the core remains perpendicular to the membrane. Overall, the different protonation of C1 exerts only a negligible perturbation on the membrane state.

The thickness and area per lipid (Table 2 and Table 3) of the membrane were calculated considering the last 25 ns of simulation. The calculated thickness for POPC-only membranes agrees with the experimental value [78,79] and indicates good membrane preparation and equilibration. Furthermore, the POPC membrane in the presence of the C1 probe at different pH shows no change in thickness, thus indicating that the molecule does not cause perturbation to the lipid medium.

The same applies to the area per lipid, whose values are shown in Table 3. As we can see, we did not have such an increase in area per lipid to say that the probe did not perfectly blend into the membrane.

**Figure 6:**
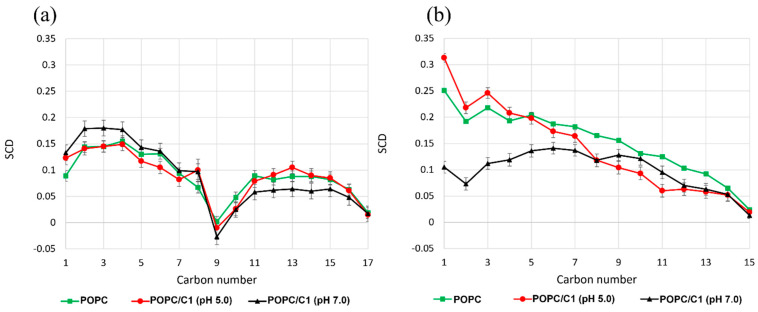
Deuterium order parameter (S_CD_) of POPC membrane without the probe C1 (green line) and with C1 at pH = 5.0 (red line) and at pH = 7.0 (black line). (**a**) Unsaturated POPC chain; (**b**) saturated POPC chain.

The S_CD_ of POPC membrane alone and in the presence of the C1 probe at pH = 7.0 and 5.0 within 5Å of the molecule of interest were compared in Figure 6. For the POPC unsaturated chain (Figure 6a), the S_CD_ decreased abruptly to ∼−0.02 in the double bond region (carbon atom 9) and approached 0 in the tail region, indicating the highest disorder within the bilayer. Similar S_CD_ profiles were observed experimentally using NMR studies that further validate our simulations [80]. In the saturated chain (Figure 6b), we observed a slight decrease in S_CD_ for phospholipids with the probe (at pH 7.0) in the initial part of the tail, indicating a higher disorder. In this case, the tricycle system was parallel to the membrane surface (see Figure 4), as shown in the density profile in Figure 5. 

Overall, SCD indicates a weak perturbation of the order and mobility of lipid chains. This supports observations of the density profile, thickness, and area per lipid of a negligible membrane perturbation of C1.

### 2.5. DFT Calculations

TD-DFT approach at DFT level, using adiabatic local density approximation and water as the simulated solvent, was used to run excitation energies calculations. Since the calculated pK_a_ of C1 was 6.3, we performed the DFT calculation of the molecules at pH 5 and 7.

Because of the rotations around the bonds among the pyridinyl moiety, the 1,3,4-oxadiazole residue, and the substituted 4-nitrophenyl moiety, 4 conformers must be taken into account for the two forms at pH 5 and 7 [81]. As shown in Figure S1, the four conformers at pH 7 do not differ significantly.

At pH 5 the protonation of the pyridinyl moiety shows a similar pattern to that seen at pH 7 without substantial differences between the 4 conformers.

**Figure 7:**
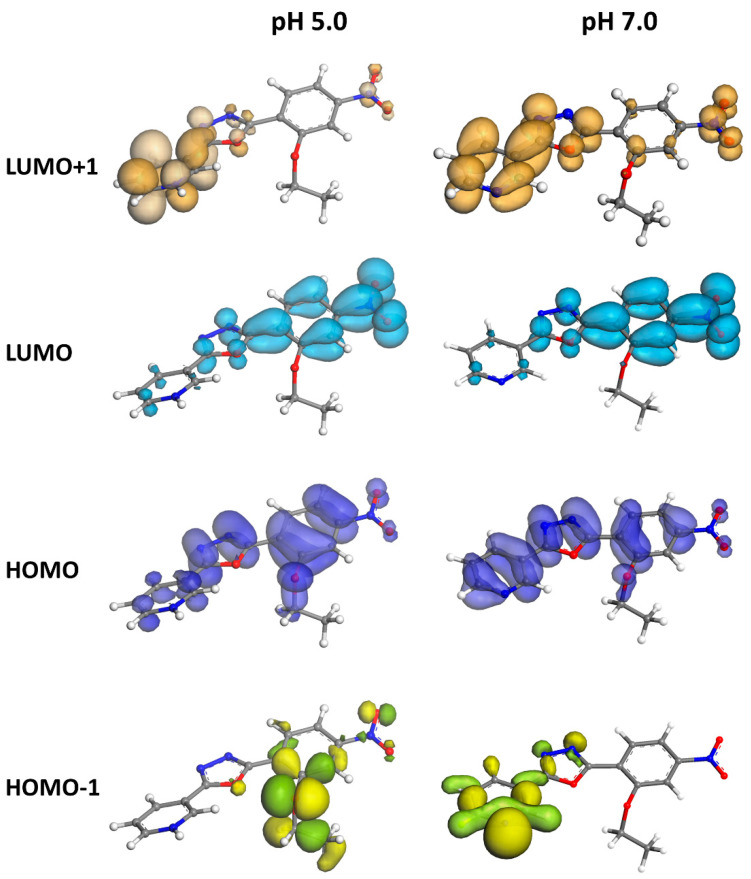
Frontier’s orbitals of C1 at pH = 5.0 (protonated form) and 7.0 (unprotonated form). Note that the tail is shortened for sake of visualization.

At pH = 7.0 the HOMO delocalization covers the entire conjugated backbone, but at pH 5.0, the protonated form of C1 has a HOMO mainly localized on the nitro group (see Figure 7). The LUMOs for both forms were similar and delocalized on two rings. The main transitions for both pH were HOMO-2 → LUMO, and HOMO → LUMO. However, HOMO → LUMO was also important, and it contributed to lower wavelengths to the absorption.

Because of the electron-withdrawing effect of the pyridinium group, at pH = 5.0 the molecule C1 showed a higher oxidation potential compared to the unprotonated form. 

In DFT calculations, we usually refer to structures relaxed in vacuum or in implicit solvent. Molecular dynamics analyses in solution have shown that the C1 molecule undergoes only a slight deviation from planarity and that all conformational changes at C1 involve rotatable bonds. Considering the presence of numerous transitions beyond the main HOMO → LUMO, the calculations are in excellent agreement with the experimental data.

**Figure 8:**
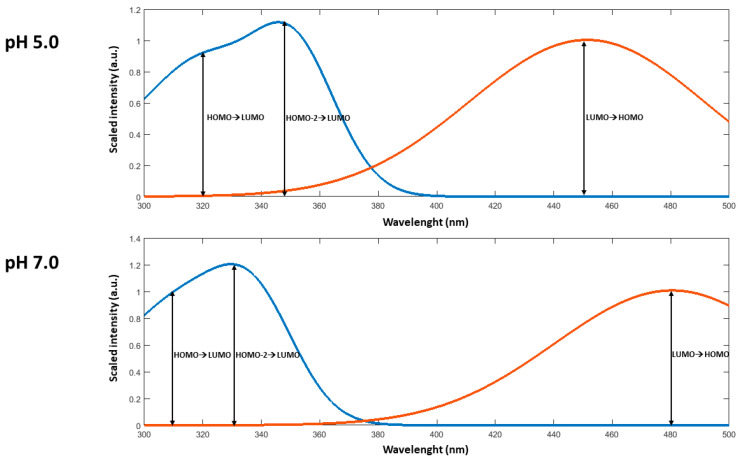
Predicted absorption (blue line) and emission (red line) spectra for C1 at pH 5.0 and pH 7.0.

The main transitions and the predicted spectra of absorption and emission are shown in Table 4 and Figure 8.

## 3. Experimental

Commercially available starting products were purchased from Sigma Aldrich. Compound (St. Louis, MO, USA) butyl 2-amino-5-hydroxy-7-(4-nitrophenyl)benzofuran-3-carboxylate was obtained as described in literature [66,82].

^1^H-NMR and ^13^C-NMR spectra were recorded in DMSO-*d*_6_ with a Bruker Advance II 400 MHz apparatus (Bruker Corporation, Billerica, MA, USA). Mass spectrometry measurements were performed using a Q-TOF premier instrument (Waters, Milford, MA, USA) with an electrospray ion source and a hybrid quadrupole-time of flight analyzer. Optical observations were performed using a Zeiss Axioscop polarizing microscope (Carl Zeiss, Oberkochen, Germany) equipped with an FP90 Mettler microfurnace (Mettler-Toledo International INC MTD, Columbus, OH, USA). The decomposition temperatures (5 wt.% weight loss), phase transition temperatures and enthalpies, were measured by DSC/TGA Perkin Elmer TGA 4000 (PerkinElmer, Inc., Waltham, MA, USA), with a scanning rate of 10 °C/min. Absorption and UV-Visible emission spectra were recorded by JASCO F-530 spectrophotometer (scan rate 200 nm min^−1^, JASCO Inc., Easton, MD, USA) and on a JASCO FP-750 spectrofluorometer (excitation wavelengths set at the absorption maxima of the samples, scan rate 125 nm min^−1^, JASCO Inc., Easton, MD, USA). Thin films of the neat samples were prepared using an SCS P6700 spin coater operating at 600 rpm for 1 min. PLQYs were calculated in water and in DMSO diluted solutions below absorbance 0.20, according to Melhuish [83].

### 3.1. Synthesis of C1

First step: synthesis of the intermediate. The intermediate 5-nitro-2-(5-(pyridin-3-yl)-1,3,4-oxadiazol-2-yl)phenol [65] was obtained by reacting 3-pyridinecarbohydrazide (1.37 g, 10.00 mmol) and 2-hydroxy-4nitrobenzoic acid (1.83 g, 10.00 mmol), which were dispersed in polyphosphoric acid (20 mL), and the mixture was stirred overnight at 150 °C. After this time, the suspension was cooled to room temperature and poured into ice/water. When the pH was regulated at about neutrality with sodium hydrogen carbonate, a whitish solid was formed, collected by filtration under vacuum and washed with water. Finally, the product was crystallized in acetone.

Second step: synthesis of the sensor C1. An amount of 2-nitro-5-(5-(pyridin-3-yl)-1,3,4-oxadiazol-2-yl)phenol, corresponding to 0.284 g (1.00 mmol) [66,82] was dissolved at 70 °C in 20 mL of tetrahydrofuran 0.413 g (1.20 mmol) of 5-bromo-N,N,N-trimethylpentan-1-aminium bromide and mixed to 0.300 mg of K_2_CO_3_, added under stirring. After 1 hour at boiling temperature, the mixture was filtered. When the solution was cooled, and the crude product precipitated in needle-like crystals. The compound was recovered and washed in methanol twice T_m_ =171 °C; T_d_= 210 °C. ^1^H-NMR (400 MHz, DMSO-*d*_6_, 25 °C, ppm): 1.56 (m, 2H), 1.81 (m, 2H), 1.93 (m, 2H), 3.05 (s, 9H), 3.29 (m, 2H), 4.38 (t, 2H), 7.73 (m, 1H), 8.04 (m, 2H), 8.34 (m, 1H), 8.48 (d, 1H), 8.87 (d, 1H), 9.23 (s, 1H). ^13^C-NMR (400 MHz, DMSO-*d*_6_, 25 °C, ppm): δ = 164.8, 162.4, 151.0, 147.3, 141.8, 137.4, 134.7, 130.2, 125.2, 124.3, 124.1, 118.7, 110.0, 67.6, 66.5, 54.9, 29.3, 25.0, 24.8 ppm. Elemental analysis calculated (%) for C_21_H_26_N_5_O_4_Br: C, 51.23; H, 5.32; N, 14.22; found: C, 51.30; H, 5.43; N, 14.16. HRMS(ESI): *m*/*z* calculated for C_21_H_26_N_5_O_4_^+^: 412.20; found 412.24 [M]^+^.

### 3.2. Single-Crystal X-ray Analysis

Crystals of C1 were obtained at room temperature by slow evaporation from an ethanol solution (about 500 μM, 2 mL). As a result, bright yellow crystals of C1 appeared as plates with typical dimensions of 0.3 mm × 0.4 mm × 0.3 mm. Data of C1 were collected with synchrotron radiation (wavelength, 0.7000 Å) from XRD1 beamline at the Elettra Synchrotron Light Source, Trieste, Italy. 

Using a small loop of fine rayon fiber, selected C1 crystals were dipped in cryoprotectant Paratone oil and flash-frozen in a stream of nitrogen at 100 K. Data was processed using XDS and POINTLESS 1.11.21 with data collection statistics reported in Table 5 [84]. No data twinning was detected. Crystals have a monoclinic unit cell with axes a = 12.20, b = 21.98, c = 9.08 Å, and space group *P* 2_1_/c (Table 5). The structure of C1 was solved by direct methods using SHELXS [85], which revealed the expected skeleton, corresponding to one molecule in the ASU and one bromide ion as counterion ensuring electroneutrality. The structure was anisotropically refined using full-matrix least-squares methods using |F^2^| against all independently measured reflections using SHELXL [86] run under WinGX suite for the refinement of small molecules [87]. No restraints were used. Hydrogen atoms were introduced and refined according to a riding model as implemented in SHELXL. Crystal data and structure refinement details for C1 are reported in Table 5. Figures were generated using Mercury CSD 3.6 [88] Crystallographic data of C1 deposited with the Cambridge Crystallographic Data Centre and can be obtained via www.ccdc.cam.ac.uk/data_request/cif (accessed on 1 March 2022)

### 3.3. Molecular Dynamics

Molecular dynamics (MD) simulations provide important information about the interaction of the C1 probe with a lipid bilayer. A model POPC membrane was constructed using YASARA Structure software [89], consisting of 180 lipids, 90 per leaflet. The membrane was simulated in the presence and absence of probe C1. Since C1 is sensitive to pH, with a color and emission change at pH = 5, simulations were performed at pH 4 and 7. The force field used was AMBER14 under NPT (normal pressure and temperature) conditions, with the Berensend thermostat. The applied cut-off was 8Å, and the particle mesh Ewald (PME) was activated to calculate electrostatic interactions. All systems were fully hydrated, respecting the water density at 0.997 g/mL, and the simulation cell was neutralized with NaCl at a final concentration of 0.9% *w*/*w*. After membrane construction and equilibration, a copy of the probe was automatically placed by the software considering the chemical nature of the molecule. Each simulation lasted for 50 ns, and the last 25 ns were used for analysis. 

#### Trajectory Analysis

The possible perturbation induced by the probe on the membrane can be predicted through MD simulations. Bilayer thickness, area per lipid, deuterium order parameter (S_CD_), and mass density profile were used to assess membrane perturbation. Membrane thickness was calculated as the average distance between the phosphorus atoms of the phospholipid heads of the two layers. The area per lipid was calculated as the total membrane area divided by the number of lipids in each leaflet. S_CD_ is an indirect measure of membrane fluidity, and it was calculated according to the Equation (1):

(1) $$S_{\mathrm{CD}}=\frac{1}{2}\;\langle 3\cos^{2}\theta_{i}-1\rangle$$

where $\theta_{i}$ is the angle between the axis given by the carbon $C_{i-1}$ and $C_{i+1}$ and the normal of the lipid bilayer. S_CD_ was calculated using VMD MembPlugin software [71].

### 3.4. DFT Calculations

Quantum-mechanical calculations were performed with the Jaguar package, Schrödinger Release 2017-4 [90], on the theoretical level DFT/B3LYP, and the molecular geometry was optimized with functional B97-D3 [91]. Charges were determined using the NBO approach. Dunning’s correlation-consistent triple-ζ basis set cc-pVTZ (-f), which includes a double set of polarization functions, was used for single-point calculations on optimized geometries. TD-DFT and Tamm–Dancoff [92] proximations were used to perform calculations at neutral compound geometry to extract absorption values from vertical excitation energies. The water solvent was simulated using Poisson Boltzmann (PB) solver [93].

The calculation of the pK_a_ of the molecule was performed with the software Jaguar [94]. The Jaguar pK_a_ prediction module calculates the pK_a_ (or pK_b_) of molecules that contain acidic or basic functional groups. The calculations involve geometry optimization of the ionic and neutral species, single-point energy and frequency calculations, and an empirical correction.

### 3.5. UV–Vis and Fluorescence pH Titrations and Naked-Eye Detection

Stock solutions (1.00 M) of Britton–Robinson buffer (at 3.0, 3.5, 4.0, 4.5, 5.0, 5.5, 6.0, 6.5, 7.0, 7.5, 8.0, 8.5, 9.0, 9.5, 10.0 pH value) were prepared in bidistilled water as described in the literature [70,95]. Aqueous solutions of C1 1.00 mM from bidistilled water were prepared for fluorescence and absorbance titrations. The titrations were performed by adding 60 µL of the buffer stock solutions to 2.5 mL of the sensor dissolved in water. The absorption or fluorescence spectra were recorded at room temperature after mixing for a few seconds. The fluorescence spectra were recorded under an excitation wavelength of 280 nm. For the naked-eye detection, buffered solutions of 1.00 mM of C1 of pH 5.0 and 7.0 were prepared, and the samples were immediately photographed in natural light and under the UV lamp operating at 254 nm.

### 3.6. In Vitro Antibacterial Activity Methods

Official standard methods were employed by UNI EN ISO accredited laboratory.

The standard UNI EN ISO 8199:2018 was followed for the preparation of the sample.

The standard UNI EN ISO 9308-1:2017 was followed to evaluate the bacterial colonies. The standard specifies a method for enumerating *Escherichia coli* and coliform bacteria based on membrane filtration, subsequent culture on chromogenic agar medium for coliforms and calculation of the number of target organisms present in the sample.

The sample preparation procedure and use of the methods for the water matrix are related to UNI EN ISO 8199: 2018 also used the formula for the colony count always in accordance with the ISO. The sample of wastewater was treated by filtering a water content of 100 mL on a nitrocellulose membrane with a porosity of 0.45 micrometers. The filtration system consists of a ramp with supports and containers (filter funnels) made of stainless steel using as suction system an electrically operated vacuum pump or a water pump. All operations are performed in sterile conditions, the supports and containers being sterilized in an autoclave or being sterile disposable. Bacteria larger than the pores of the filter, remain trapped on the surface of the filter itself. After filtration, each membrane is placed in a plate containing a specific culture medium that allows the growth and differentiation of the bacteria sought. The media used were prepared in accordance with ISO 11133: 2018, respecting the conditions of selectivity, specificity, and sterility. In our case, the medium used for *Escherichia coli* and for total coliforms was chromogenic coliform agar. The plates prepared with the substance to be tested at the known concentrations were incubated at a temperature of 37 °C for 24 h +/− 2 h for both the investigated microorganisms. After the incubation time, the results were read by comparing the plates used for the blank or without substance and those with the addition, therefore evaluating a decrease in the number of colonies.

## 4. Conclusions

A novel multifunctional probe based on 2-phenyl-5-(pyridin-3-yl)-1,3,4-oxadiazole skeleton with a flexible cationic chain was designed, synthesized, and structurally characterized. The colorimetric and fluorescent response of C1 to pH parameter was explored by naked-eye analysis and proton titration experiments. A colorimetric “turn-on” and a fluorescent “turn-off” was detected in the range 5.5–6.5. Antimicrobial activity measured in vitro showed C1 ability against *Escherichia coli*, preserving the antimicrobial activity showed by its scarcely soluble precursor. Single-crystal X-ray study gave information about the structure of C1, and DFT calculations were used to rationalize the spectroscopic response. Molecular dynamics simulations of the interactions of C1 with a model POPC membrane demonstrated the potential to cross the lysosomal membrane with a low degree of bilayer perturbation.

In summary, the introduction of an amphiphilic chain on a suitable aromatic scaffold can direct and even improve desired properties, achieving a multifunctional probe including good solubility in organic and in aqueous media, improved optical response in diluted water solution, and antimicrobial activity.

## Figures and Tables

**Table 1:** Absorption and emission maxima of C1 in slightly acidic (pH = 5.0) and neutral (pH = 7.0) solutions.

Sample	λ_ab_^(a)^	λ_em_^(b)^	PLQY% ^(e)^	λ_ab_^(c)^	λ_em_^(d)^	PLQY% ^(e)^
C1	Water			DMSO		
pH = 5.0	291	467	3.0	292	486	12.5
pH = 7.0	290	508	0.4	292	547	3.1

^(a)^ Wavelength of UV-visible absorption maxima [nm] in water, ^(b)^ wavelength of emission maxima [nm] in water; ^(c)^ wavelength of UV-visible absorption maxima [nm] in DMSO; ^(d)^ wavelength of emission maxima [nm] in DMSO; ^(e)^ emission PLQYs in solutions.

**Table 2:** Thickness of a POPC membrane in the absence and presence of the C1 probe at different pH values.

POPC (Exp)	POPC	POPC/C1 (pH = 5.0)	POPC/C1 (pH = 7.0)
39.8 ± 0.8	37. 5 ± 0.3	37.7 ± 0.2	37.5 ± 0.2

**Table 3:** Area per lipid of a POPC membrane in the absence and presence of the C1 probe at different pH values.

POPC (Exp)	POPC	POPC/C1 (pH = 5.0)	POPC/C1 (pH = 7.0)
68.3	70.3 ± 0.5	68. 6 ± 0.6	69.2 ± 0.4

**Table 4:** Computed absorption and emission maxima of C1 in slightly acidic (5.0) and basic (7.5) solutions.

Sample	λ_ab_^(a)^	λ_em_^(b)^
C1		
pH = 5.0	350 (HOMO-2→LUMO) 320 (HOMO→LUMO)	451 (LUMO→HOMO)
pH = 7.0	336 (HOMO-2→LUMO) 310 (HOMO→LUMO)	485 (LUMO→HOMO)

Wavelength of ^(a)^ UV-Visible predicted absorption and ^(b)^ emission maxima [nm] in water.

**Table 5:** Data collection and refinement statistics of C1 compound.

	C1
CCDC number	2067327
Asymmetric unit content	C1•Br•H_2_O
Color	Bright yellow
Temperature (K)	100
Wavelength (Å)	0.7000
Crystal system	Monoclinic
Space group	*P* 2_1_/c
*a* (Å)	12.204 (2)
*b* (Å)	21.977 (4)
*c* (Å)	9.075 (2)
β (°)	107.19 (3)
Theta (°)	30.2–1.7
R-merge	0.118
I/s(I)	11.2
Completeness (%)	94.3
Multiplicity	6.5
Mosaicity (°)	0.26
Volume	2325 Å^3^
Z	1
Calculated density	1.20 g/cm^3^
*q* max (°)	29.746
Reflections unique	4081
Data/restraints/parameters	4081/0/301
*R1* indices (I > 2s(I))	0.0625 (0.103, all data)
*wR2*	0.219 (0.193, all data)
*F*_000_	862
Largest diff. peak and hole	0.48 and −0.63 e-/Å^3^
Goodness-of-fit on |F^2^|	1.01

## Data Availability

Data is contained within the article or Supplementary Materials.

## Supplementary Materials

The following supporting information can be downloaded at: https://www.mdpi.com/article/10.3390/molecules27061824/s1, Figure S1. HOMO (blue and light blue) and LUMO (yellow and orange) orbitals for the four conformers of C1 (a, b, c, d) at pH 7; Figure S2. Close up of the UV-Vis absorption spectra of C1, as reported in Figure 1 (left side).

## Sample Availability

Samples of the compounds C1 is available from the authors.
