# Supplementary material for: A Water Soluble 2-Phenyl-5-(pyridin-3-yl)-1,3,4-oxadiazole Based Probe: Antimicrobial Activity and Colorimetric/Fluorescence pH Response
Source: Molecules. 2022 Mar 11;27(6):1824. doi: 10.3390/molecules27061824 (PMC8952330; doi:10.3390/molecules27061824)
Supplement: Supplementary file 1 [file molecules-27-01824-s001.zip › molecules-1608393-supplementary.pdf]

A Water Soluble 2-Phenyl-5-(pyridin-3-yl)-1,3,4-oxadiazole Based Probe. Antimicrobial Activity and Colorimetric/Fluorescence pH Response

Rosita Diana[a], Ugo Caruso[b*], Luigi Di Costanzo[a], Simona Concilio[c], Stefano Piotto[c], Lucia Sessa[c] and Barbara Panunzi[a]

[a] Department of Agriculture, University of Napoli Federico II, Via Università, 100 - 80055 - Portici (NA), Italy
[b] Department of Chemical Sciences, University of Napoli Federico II, Strada Comunale Cinthia, 26, 80126 Napoli, Italy
[c] Department of Pharmacy, University of Salerno, Via Giovanni Paolo II, 132, 84084 Fisciano (SA), Italy

Supplementary Material

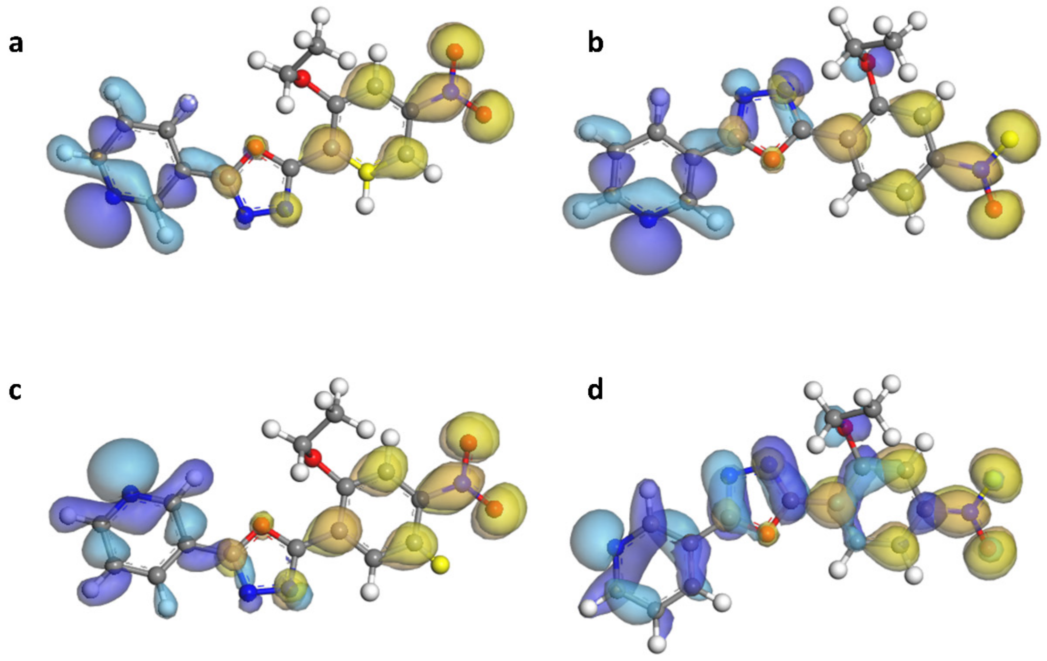

Figure S1. HOMO (blue and light blue) and LUMO (yellow and orange) orbitals for the four conformers of C1 (a, b, c, d) at pH 7.

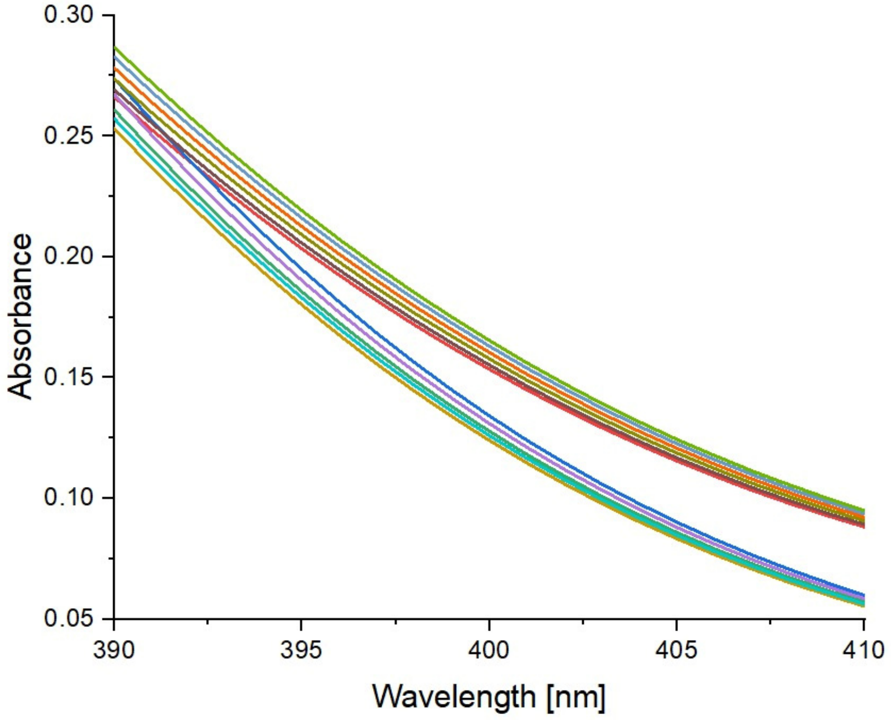

Figure S2. Close up of the UV-Vis absorption spectra of C1, as reported in Figure 1 (left side)
